# A self-assembled nanoparticle vaccine elicits effective neutralizing antibody response against EBV infection

**DOI:** 10.3389/fimmu.2024.1530364

**Published:** 2025-01-03

**Authors:** Ping Li, Ziyi Jiang, Jingjing Shi, Haochuan Sha, Zihang Yu, Yan Zhao, Sanyang Han, Lan Ma

**Affiliations:** ^1^ Institute of Biopharmaceutical and Health Engineering, Tsinghua Shenzhen International Graduate School, Tsinghua University, Shenzhen, China; ^2^ Institute of Biomedical Health Technology and Engineering, Shenzhen Bay Laboratory, Shenzhen, China; ^3^ College of International Education, Henan University of Technology, Zhengzhou, China; ^4^ Institute of Bio-Architeture and Bio-Interactions, Shenzhen Medical Academy of Research and Translation, Shenzhen, China; ^5^ State Key Laboratory of Chemical Oncogenomics, Tsinghua Shenzhen International Graduate School, Tsinghua University, Shenzhen, China

**Keywords:** Epstein-Barr virus (EBV), vaccine, epitope, ferritin, nanoparticle

## Abstract

**Background:**

Epstein–Barr virus (EBV) is a significant global public health concern because of its association with various malignancies and autoimmune diseases. Over 90% of the global population is chronically infected with EBV, impacting numerous cancer-related cases annually. However, none of the effective prophylactic vaccines against EBV is approved at present.

**Methods:**

In this study, we developed a novel vaccine candidate based on epitope peptides from the receptor-binding domain of EBV-encoded gp350 glycoprotein to prevent EBV infection. These epitope peptides detected a binding capability with host cells were then fused by flexibility linkers and expressed in *Escherichia coli* to reduce the unnecessary glycan modifications to simulate their free-glycan status. The fused recombinant protein (L350) was displayed on the surface of ferritin-based nanoparticle. The immunogenicity of the L350–ferritin nanoparticle was evaluated in Balb/c mice, and the neutralizing titers of sera from immunized mice were detected by means of an infection blocking assay in an *in vitro* cell model.

**Results:**

All the five epitope peptides could bind to AKATA cells, and their fused recombinant protein (L350) was successfully presented on the surface of self-assembled ferritin nanoparticles. Sera from the L350–ferritin nanoparticle-immunized mice showed high titers of both L350 protein-specific and gp350D_123_ protein-specific antibodies, and sera from gp350D_123_ protein-immunized mice could also recognize L350 protein well. Most importantly, the L350–ferritin nanoparticle induced efficient neutralizing antibodies to block EBV-GFP infection in AKATA cells and also constructed a strong antigen-specific B-cell memory in immunized mice. Moreover, histopathological changes of main tissues from all vaccinated mice were not observed.

**Conclusion:**

These data indicate that the L350–ferritin nanoparticle vaccine candidate has considerable potential application in preventing EBV infection and provides a promising basis for developing prophylactic EBV vaccines.

## Introduction

Epstein–Barr Virus (EBV), a member of the gamma herpesvirus family, is the first virus identified as an oncogenic virus in human, and more than 90% of the population is chronically infected globally (1). Although primary EBV infection is usually asymptomatic or manifests with mild symptoms, a subset may develop into serious EBV-associated diseases, such as Hodgkin’s lymphoma (HL), Burkitt’s lymphoma (BL), NK/T cell lymphoma, post-transplant lymphoproliferative disease (2), and malignancies like nasopharyngeal carcinoma (NPC) and gastric carcinoma (GC) (3, 4). EBV-associated tumorigenesis is usually driven by EBV-encoded proteins and its genome in latent infected cells, causing complex dysregulations and epigenomic aberrations, such as DNA hypermethylation, epigenomic rewiring, and enhancer dysregulation, and finally leading to cancer progression (5, 6). Every year, approximately 200,000 EBV-associated cancer cases were reported globally (7, 8), causing serious threat to human life and health. The most effective method to prevent EBV-associated cancer is prophylactic vaccine, which blocks EBV infection at the origin. However, none of the vaccines against EBV infection and EBV-associated disease is approved at present.

The envelope glycoproteins of EBV were well identified, providing significant foundation for the research and development of prophylactic vaccines. The most abundant glycoprotein of EBV is gp350 glycoprotein, a key target of prophylactic vaccines with an ability to neutralize B-cell infection (9, 10). Other glycoprotein fusion apparatuses including gH/gL, gH/gL/gp42, and gB were also reported to reduce neutralizing antibodies against EBV infection (11). To provide more complete protection, vaccine-fused multiple and necessary glycoproteins is indeed needed, and then the virus-like particles (VLPs) and nanoparticle vaccines were applied. Recently, nanoparticle vaccines displaying multiple immunogens were reported to induce cross-reactive B-cell responses, providing an avidity advantage over strain-specific B-cell receptor (BCR) interactions that were incapable of facilitating bivalent binding (12). In addition, nanoparticle vaccines displayed some significant advantages, such as more efficient antigen presentation, higher specific antibody titer, and stronger protection (13–16). These studies promoted the development of nanoparticle vaccines, especially for displaying multiple immunogens.

The gp350 protein is extensively post-translationally modified by both N- and O-linked glycosylation (17). The encapsulation by high-density glycans not only avoids recognition by immune response (18) but also provides difficulty for vaccine design. The glycan-free surface of gp350 protein was identified as its receptor-binding domain (RBD) (19–21), which was directly recognized and bonded by the monoclonal antibody 72A1, a well-studied neutralizing antibody against EBV by blocking the binding of gp350 protein to its receptor CR2 (22, 23). Subsequently, studies aimed to investigate and simplify the effective epitopes from gp350-RBD (24, 25), and these epitopes displayed the ability to elicit antibodies blocking EBV infection (26, 27). These epitope peptides act as critical functional domains for receptor recognition and then mediate viral adhesion and invasion. Thus, these epitope peptides could elicit neutralizing antibody to block viral infection. Epitope peptide-based vaccines could provide more neutralizing epitopes, compared with full-length or truncated protein antigens under equal quality, thus inducing a stronger neutralizing antibody response. However, epitope peptides showed lower immunogenicity because of their low molecular weight. Fortunately, nanoparticle-based polymerization helped to enhance their immune responses and addresses this limitation effectively (28–31).

Ferritin is a highly conserved family of supramolecular nanostructures encoded by various organisms and can self-assemble into a hollow 24-mer spherical structure with a diameter of approximately 12 nm (14). Because of its excellent biocompatibility, ferritin is widely used in research applications such as biological detection, imaging diagnosis, and drug delivery (32). Here, we used *Helicobacter pylori-*derived ferritin as a fusion apparatus to display antigen proteins on its surface to construct nanoparticle vaccine. We designed four surface-exposed peptides based on EBV gp350-RBD and a peptide with neutralizing ability outside the region. These peptides were investigated to bind with the AKATA cells, an EBV-negative BL originated cell line, and then were fused as a recombinant protein and arranged according to their spatial distribution on the gp350 protein. It was expressed in *Escherichia coli* to reduce the post-translated glycan modifications to simulate the free-glycan status of these epitopes. The fused recombinant protein (L350) was assembled on the ferritin nanoparticle by the irreversible binding of SPY tag on L350 and CATCHER on ferritin. Through size-exclusion chromatography, the L350–ferritin nanoparticles were purified and then immunized in Balb/c mice to evaluate their immunogenicity and safety. Interestingly, the sera from L350–ferritin nanoparticle vaccine-immunized mice elicited a high titer of gp350D_123_ protein-specific antibody, and sera from gp350D_123_ protein could also recognize L350 protein with high titer. More importantly, these sera samples could block EBV infection in AKATA cells *in vitro*, demonstrating their neutralization capacity. Moreover, the L350–ferritin nanoparticle vaccine generated strong gp350D_123_ protein-specific B-cell memory in immunized mice and showed little histopathological changes. Together with these data, the L350–ferritin nanoparticle vaccine, a ferritin nanoparticle displaying gp350 protein-originated epitope peptide-fused antigens, induced potent neutralizing antibody response against EBV infection, and the L350–ferritin nanoparticle vaccine candidate provides a promising base for developing prophylactic EBV vaccines.

## Materials and methods

### Synthesis of peptides and construction of plasmids

The peptides used in this study were synthesized by GL Biochem Ltd. The sequences of recombinant protein were optimized for codon usage and synthesized by RayBiotech, and the L350 and ferritin were cloned into pET-28a plasmid for expression in *E. coli*. The sequences of gp350D_123_ glycoprotein were optimized for codon usage and cloned with an IL-2 secreted peptide at the N-terminus, and a 6×His tag at the C-terminus into the lentiviral vector pLVX-CMV-IRSE-GFP. The plasmid was confirmed by sequencing.

### Cell culture

AKATA lymphocytes and CNE2-EBV-GFP cells were kindly gifted by Prof. Yixin Zeng at the Sun Yat-sen University Cancer Center. These cells were cultured in 1640 medium (Gibco) supplemented with 10% fetal bovine serum (FBS, Gibco). HEK293F cells were obtained from the Cell Bank of the Chinese Academy of Sciences and were cultured in specific cell culture medium (OPM-AM412, OPM). All cells were maintained at 37°C in a humidified incubator with 5% CO_2_.

### Protein expression and purification

HEK293F cells were transfected with lentiviral plasmid for gp350D_123_ protein expression. Approximately 72 h post-transfection, the cell supernatant was collected and filtered by a 0.45-μm filter for purification. The recombinant ferritin protein and L350 protein were expressed in *E. coli* BL21(DE3) cells. Transformed bacteria were cultured in LB medium containing kanamycin (50 µg/mL) at 37°C until the OD_600_ nm reached 0.6–0.8 and induced with 0.4 mM isopropyl-b-D-thiogalactopyranoside (IPTG) at 16°C for 18 h and then were suspended with lysis buffer (50 mM tris-HCl, pH 7.5, 500 mM NaCl, 5% glycerol, and 1 mM DTT) and incubated with lysozyme on ice for 30 min, and then sonicated on ice. The lysate was filtered by a 0.45-μm filter and purified by Ni-NTA (GE healthcare) in an AKATA Protein Purification System. The His-tag protein was eluted with elution buffer (50 mM tris-HCl, pH 7.5, 500 mM NaCl, and 300 mM imidazole) and further dialyzed in PBS overnight at 4°C. The protein was further purified in a Superose 6 Increase 10/300 GL gel filtration column (GE healthcare), and the protein concentration was determined using the BCA protein assay kit (Solarbio).

### SDS-PAGE and Western blotting

The cell samples and protein samples were boiled in loading buffer. Samples were subjected and separated by SDS-PAGE electrophoresis and transferred to PVDF membranes (Millipore). PVDF membranes were blocked in 5% bovine serum albumin (BSA) and incubated with the corresponding antibodies. The whole SDS-PAGE gel could be stained by Coomassie blue buffer for the analysis of protein purity.

### Nanoparticle self-assembly and purification

The purified ferritin protein and recombinant L350 protein were mixed in PBS at a molar ratio of 1:10 and incubated at 4°C overnight. The assembled nanoparticle in the mixture was purified using a Superose 6 Increase 10/300 GL gel filtration column (GE Healthcare). The protein solution was then concentrated by an ultrafiltration device (100-kDa cutoff). The concentration was determined by the BCA protein assay kit (Solarbio).

The nanoparticle size was further analyzed by dynamic light scattering (DLS). Briefly, the intensity of freshly synthesized liposome and gp350D_123_-conjugated liposome samples was measured at a scattering angle of 90° using a Nanoparticle Size Analyzer (Nano ZS90, Malvern, UK).

### Transmission electron microscopy

The nanoparticles were analyzed by negative staining electron microscopy. Briefly, samples were diluted to 0.5 mg/mL and applied to 200-mesh carbon-coated copper grids for 5 min. The excessive solution on the grids was removed, and the grids were washed three times by double-distilled water. Then, the negative staining was performed immediately with freshly filtered 2% phosphotungstic acid (pH 6.4) for 30 s. Grids were imaged with a FEI Tecnai T12 TEM (FEI, USA) at an accelerating voltage of 120 kV and photographed at a magnification of 25,000-fold.

### Fluorescent labeling and purification of peptides and proteins

The proteins and peptides were labeled with NHS-Biotin or NHS-APC according to the instructions of labeling kits (Elabscience). Briefly, proteins or peptides (1 mg) were placed into an appropriately sized ultrafiltration device, and the volume was adjusted to 0.5 mL with Labeling buffer II, and then concentrated by centrifugation at 12,000*g* for 10 min. The samples in the filter device were recycled and Labeling buffer II was added into the samples to a final concentration of 2 mg/mL, and then 9.8 μL of NHS-Biotin/NHS-APC (12.31 mM) was mixed into the sample and incubated at 37°C in the dark for 30 min. Subsequently, 1 M Tris-HCl (pH 8.7) was added into the mixture, in 10 μL per 100 μg of proteins or peptides, and incubated at room temperature for 10 min. PBS was added into the samples to the total volume to 0.5 mL. The solution was gently mixed and transferred to an appropriately sized ultrafiltration device for ultrafiltration and centrifugation. The labeled proteins and peptides were collected.

### Peptide cell binding assay

The peptide cell binding assay was performed as precious reported (24). Briefly, the Biotin-labeled peptides were diluted to a final concentration of 1 μM with PBS. Approximately 10^6^ AKATA cells were mixed with the labeled peptides at concentration gradients of 2.5, 5.0, 10, 20, and 50 nM and then incubated at 18°C for 1 h. Then, cells were stained with FITC-conjugated streptavidin for 30 min. The cells were isolated by centrifuging at 1,000*g* for 5 min and washed five times with PBS. The peptide cell binding was detected by flow cytometry. The gating strategies are described in figure legends.

### Ethics statement and mouse immunization assay

All experiments involving mice were approved by the Institutional Animal Care and Use Committee at the Tsinghua Shenzhen International Graduate School, and the animals were cared for in accordance with the institutional guidelines. All the mice were purchased from ZhuHai Bestest Biotechnology Co., Ltd.

Specific pathogen-free (SPF) female Balb/c mice (6 weeks) were immunized by subcutaneous (s.c.) injection of six mice per group. Approximately 15 μg of purified proteins in 100 μL of PBS was injected. The primary injection was performed at week 0, and two booster injections were given at 2-week intervals (weeks 2 and 4). The serum samples of immunized mice were collected at weeks 0, 2, 4, 6, 8, 10, 12, 14, and 16 for testing IgG titers and neutralization antibody titers. All serum samples were stored at −80°C prior to use. Euthanasia was performed at week 16, and the major tissues including heart, liver, spleen, lungs, and kidneys were collected for histopathology analysis, and the spleen of each immunized mice was collected for splenocyte isolation.

### Splenocyte isolation and cell staining

The isolation of splenocytes was performed as described previously (13). Briefly, the spleen was collected and washed five times with sterilized PBS and then homogenized through a 70-μm pore size cell strainer. The cells were suspended and incubated in ACK lysis buffer to remove red blood cells (RBCs). To stain the surface markers of B cells, splenocytes were stained with APC-labeling gp350D_123_ protein and fluorescence-conjugated monoclonal antibodies (B220-PE, CD38-FITC, Elabscience) for 30 min in PBS (containing 0.5% BSA) on ice. The cells were isolated by centrifuging at 1,000*g* for 5 min and washed five times with PBS and then analyzed by flow cytometry (Beckman). The gating strategies for splenocytes were described in figure legends.

### Indirect enzyme-linked immunosorbent assay

Purified gp350D_123_ protein was coated on 96-well microplates (Jet Bio) (200 ng/100 μL/well) in CBS buffer (0.015 M Na_2_CO_3_ and 0.035M NaHCO_3_, pH 9.6) at 4°C overnight. The plates were washed three times and then blocked with blocking buffer (0.02 M PBS, pH 7.4, containing 0.05% Tween20 and 1% BSA) for 1 h at 37°C. The plates were washed three times and then 10-fold serial diluted sera samples were added and incubated for 1 h at 37°C. The plates were washed three times and 100 μL of horseradish peroxidase (HRP)-conjugated goat anti-mouse IgG was added (Solarbio) (1:4,000 dilution) to incubate for 1 h at 37°C. Signals were developed in buffer (0.0243 M citric acid, 0.0514 M Na_2_HPO_4_, 0.045% H_2_O_2_, and 0.0037 Mo-phenylenediamine) for 10–20 min. The reaction was stopped by 2 mol/L H_2_SO4. The absorbance was measured at 492 nm within 5 min by using a microplate reader (Molecular Devices). The cutoff value was set according to the value of negative samples (blocking buffer and pre-immune sera).

### Induction and purification of EBV-GFP virus

The CNE2-EBV-GFP cells were cultured in 10-cm dishes with an additional final concentration of 100 μg/mL G418. After approximately three passages, the viral production was performed as previously reported (33). Approximately 12 h after cell passage, CNE2-EBV-GFP cells were treated with 20 ng/mL 12-o-tetradecanoylphorbol 13-acetate (TPA) and 2.5 mM sodium butyrate for 24 h to activate EBV genome from the latent phase into the lytic cycle. After culturing in fresh medium for 72 h, the supernatant was collected and centrifuged at 3,000*g* for 10 min and then filtered by a 0.8-μm filter. The filtered supernatant was concentrated and purified by ultracentrifugation at 50,000*g* for 2.5 h at 4°C. The white pellet, containing viral particles, was collected and gently resuspended in 1640 medium to store at −80°C for later use.

To infect AKATA cells (EBV negative), the EBV-GFP was diluted with 1640 medium and then added to 10^4^ AKATA cells per well in 96-well plates for 2 h at 37°C. The cells were washed with PBS and incubated for a further 36 h in fresh medium. The EBV-GFP infection rate was detected by calculating the percentage of GFP-expressing cells (GFP+) with flow cytometry and the results were analyzed by FlowJo software.

### Infection blocking assay

The EBV-GFP infection assay was performed as previously reported (34). Serum samples collected from immunized Balb/c mice were serially diluted (starting from 1/2 to 1/512), and mixed with 50 μL of virus (in RPM1640) to incubate for 2 h at 37°C. Subsequently, these incubated samples were added to 10^5^ AKATA cells (EBV negative) at 37°C for 3 h. After incubation, the cells were pelleted by centrifugation and washed once using PBS before being cultured in RPM1640 with 10% FBS in 24-well plates for 48 h. The cells were then collected and washed three times with PBS. The EBV infection rate was determined by measuring the production of GFP+ cells by flow cytometry. In this assay, uninfected cells were used as negative controls and AKATA-negative cells incubated with EBV in the absence of antibodies were used as positive controls.

### Histopathology analysis

Vaccine-immunized Balb/c mice were euthanized, and their major tissues including heart, liver, spleen, lungs, and kidneys were collected to fix in 4% paraformaldehyde and then treated by embedding with paraffin. Tissues were cut into longitudinal sections (3–4 μm) and then were stained with hematoxylin and eosin (H&E). Images were captured with Pannoramic MIDI (3DHISTECH Ltd).

### Statistics

The details of statistical analysis are described in each figure legend. These data are shown as the mean ± SD. The statistical significance between different groups was calculated by two-tailed unpaired Student *t-*test. *p*-value < 0.05 was significant. Data are analyzed with the GraphPad Prism 8.3 software.

## Results

### Immunogen design based on gp350 RBD-originated epitope peptides

**Figure 1:**
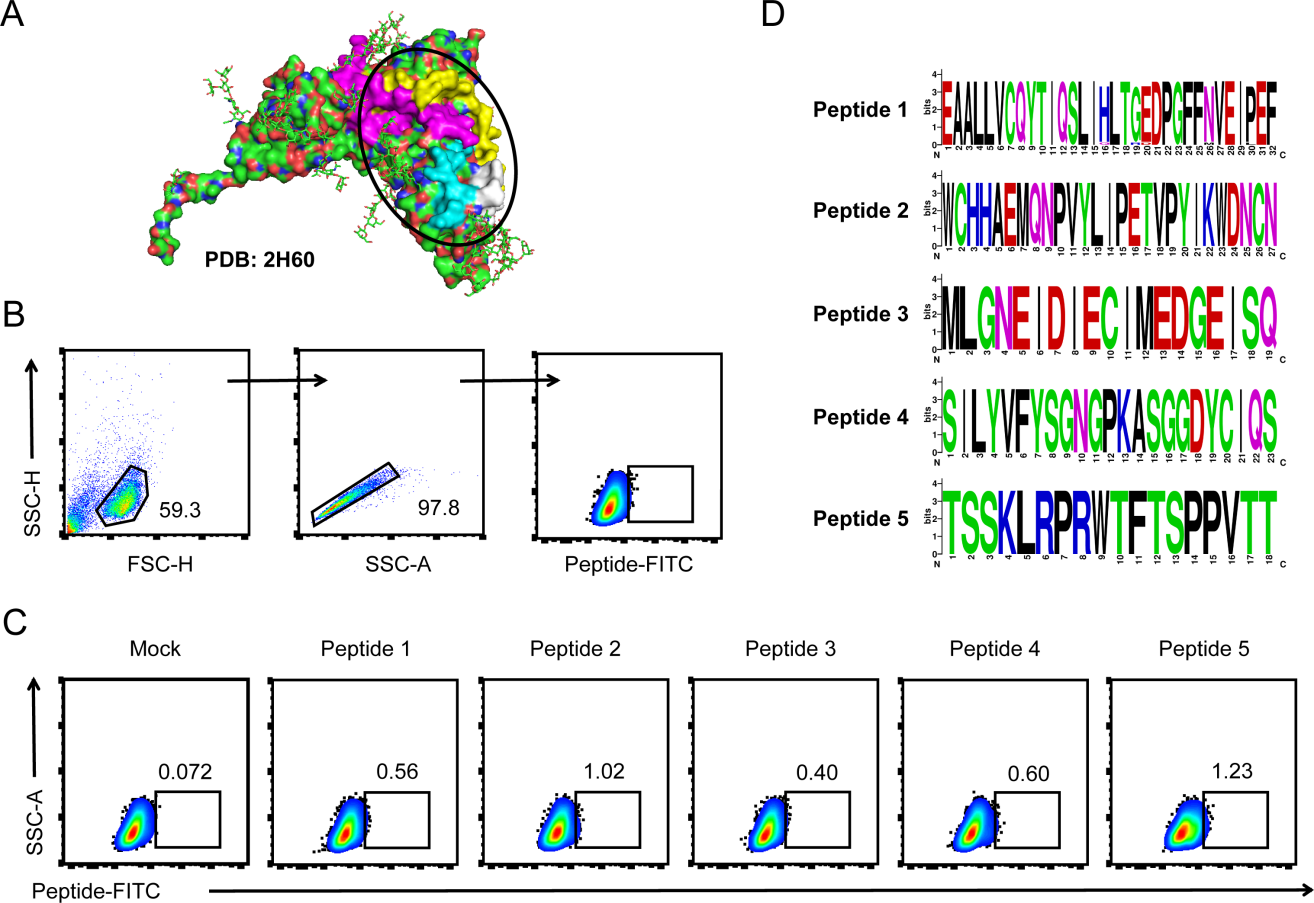
EBV-encoded gp350 glycoprotein-originated epitope peptides recognized and bound with host cells. **(A)** The structure of EBV-encoded gp350 glycoprotein (PDB: 2h60) was visualized using PyMOL software, and regions interacting with the CD21/CR2 receptor were circled out as receptor binding domain (RBD). **(B, C)** The binding of epitope peptides to AKATA cells was detected by flow cytometry. Gating strategy for FITC-peptide binding cells **(B)**. All five candidate peptides were labeled by NHS-Biotin and incubated with AKATA cells and then stained with FITC-conjugated streptavidin, and a peptide derived from an intracellular protein was set as the negative control **(C)**. **(D)** Conservancy analysis of 50-amino-acid sequences of EBV-encoded gp350 glycoprotein-originated peptides was performed using the MEGA11.0.13 software (50 sequences per peptide), and the results were displayed using WebLogo.

Structural studies have resolved the co-crystal structure of gp350 glycoprotein with its cell surface receptor CD21/CR2. In this study, we used PyMOL software to display the structure of the gp350 glycoprotein sequence PDB 2h60, and the binding domain with the CD21/CR2 receptor was highlighted (**Figure 1A**). We selected four peptides in the RBD and a peptide in another domain of gp350 protein with minimal glycan coverage as candidate peptides. We firstly investigated the binding of peptides to AKATA cells. These Biotin-labeled peptides were purified to incubate with AKATA cells and stained by FITC-conjugated streptavidin. The percentage of FITC-positive cells were detected by flow cytometry (**Figure 1B**). As shown in **Figure 1C**, these five candidate peptides could bind to the cells, and peptide 2 and peptide 5 showed higher binding rates, compared with a control peptide derived from an intracellular protein. Finally, to analyze the conservation of these five peptides in different EBV strains, we downloaded 50 amino-acid sequences of gp350 glycoprotein from NCBI and analyzed them using the MEGA11.0.13 software, and the results showed high conservation among these strains (**Figure 1D**). These data indicated that the five peptides possessed the ability to mediate the binding of EBV virions to AKATA lymphocytes and conservation in EBV strains.

### Construction of L350–ferritin nanoparticles based on epitope peptides from EBV-gp350 glycoprotein

**Figure 2:**
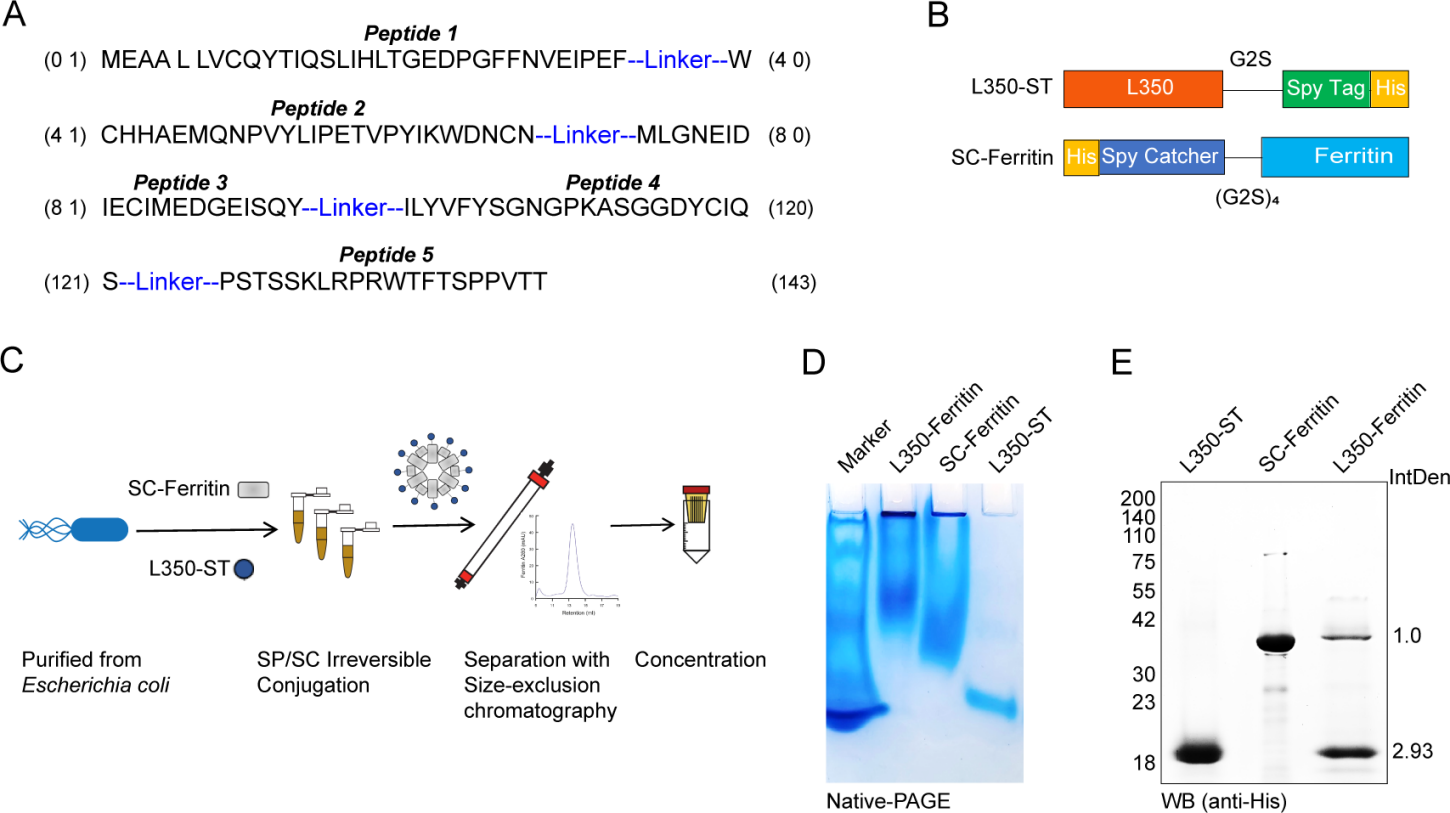
Schematic diagram of the rational design and peptide combination vaccine candidates. **(A)** The five peptides were concatenated according to their distribution in EBV-encoded gp350 glycoprotein with a flexible linker (GGGGS) to conduct a fused recombinant protein L350. **(B)** The SPY tag and 6×His tag were cloned at the C-terminus of the fused L350 protein (L350-ST), and a Catcher tag and a 6×His tag were cloned at the N-terminus of the ferritin protein (SC-Ferritin). **(C)** The schematic diagram of the rational design of L350–ferritin nanoparticles. The L350-ST and SC-Ferritin protein were expressed in *Escherichia coli* and purified, and these purified proteins were incubated to self-assemble, and the assembled L350–ferritin nanoparticles were purified using a size-exclusion chromatography column (Superose 6 Increase 10/300 GL), and then concentrated using a 100-kDa cutoff ultrafiltration device. **(D)** The native-PAGE assay detecting the assembly of nanoparticles. **(E)** Western blot detection of the composed purified L350–ferritin nanoparticles, and the protein level was quantified by using the ImageJ software.

In this study, the five peptides were concatenated in the order of their distribution from the N-terminus to the C-terminus on the gp350 glycoprotein and connected by a flexible linker (GGGGS) to conduct a fused recombinant protein, named L350 (**Figure 2A**). A SPY tag and a 6×His tag were cloned at the C-terminus of the L350 protein (L350-ST), and a Catcher tag and 6×His tag were cloned at the N-terminus of the ferritin protein (SC-ferritin) (**Figure 2B**). The Catcher protein could specifically recognize the SPY tag and form irreversible binding under mild conditions, thereby anchoring the L350-ST recombinant protein to the ferritin nanoparticle surface. The L350 protein and ferritin protein were cloned into pET-28a plasmids and expressed in *E. coli* to reduce post-translational glycan modifications. Subsequently, the L350-ST protein and SC-ferritin protein were incubated at 4°C overnight, and the fully assembled L350–ferritin nanoparticles were further separated and purified by size-exclusion chromatography and concentrated by an ultrafiltration device with a 100-kDa cutoff (**Figure 2C**). The L350–ferritin nanoparticles purified by size-exclusion chromatography showed a larger size with a retention volume at 10.2 mL, compared with ferritin nanoparticles at 13.5 mL. Then, the assembly of ferritin and L350–ferritin nanoparticles was further detected by a native-PAGE with Coomassie blue staining. As shown in **Figure 2D**, the mobility of these ferritin and L350–ferritin nanoparticles was apparently retarded, indicating the assembly of ferritin nanoparticle and the formation of an L350–ferritin complex. In addition, these purified L350–ferritin nanoparticles were detected by Western blot and the results showed that the L350–ferritin was composed of ferritin and L350 protein, with an average integrated density (IntDen) of the two protein level of 1:2.93 (**Figure 2E**). These data demonstrated the sufficient assembly of L350 protein to ferritin nanoparticles.

**Figure 3:**
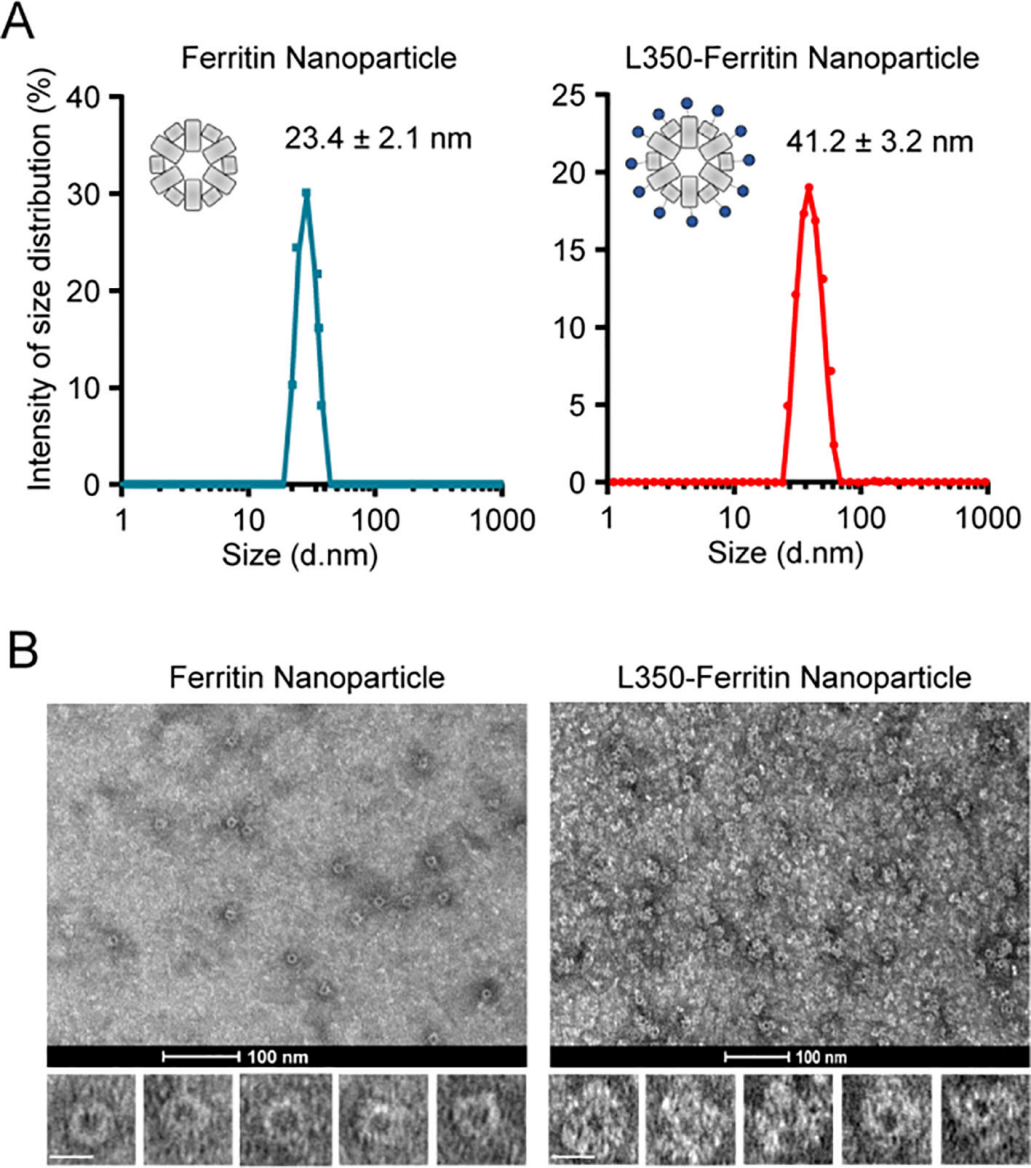
Characterization of self-assembly of the fusion antigens into ferritin nanoparticles. **(A)** Dynamic light scattering (DLS) analyzing the particle size of the purified ferritin nanoparticles and L350–ferritin nanoparticles. The curves of the particle size were drawn by GraphPad Prism 8.3 software. Ferritin nanoparticles (Blue), L350–ferritin nanoparticles (red). *n* = 3 independent repeats. Data are mean ± SD. **(B)** Transmission electron microscopy (TEM) analyzing the shape of the purified ferritin nanoparticles and L350–ferritin nanoparticles. Scale bars represent 100 nm (upper) and 20 nm (lower).

Further analysis of the purified L350–ferritin nanoparticles by DLS revealed their particle size. The results showed that both the ferritin nanoparticles and L350–ferritin nanoparticles exhibited a single peak in particle size, with diameters of 23.4 ± 2.1 nm and 41.2 ± 3.2 nm, respectively (**Figure 3A**). The particle size of the L350–ferritin nanoparticles showed a substantial increase, indicating the conjugation of L350 protein into the ferritin nanoparticle. Furthermore, transmission electron microscopy (TEM) was used to characterize the shape of the nanoparticles. Compared with the ferritin nanoparticle, L350–ferritin nanoparticles had a larger particle size, consistent with the results of DLS measurements. In addition, in contrast to the smooth surface of ferritin nanoparticles, the L350–ferritin nanoparticles exhibited a relatively rough surface, indicating successful assembly of L350 protein on their surface (**Figure 3B**). These results indicated the successful preparation of the L350–ferritin nanoparticle vaccine.

### The L350–ferritin nanoparticle vaccine induced potent humoral immune responses in Balb/c mice

**Figure 4:**
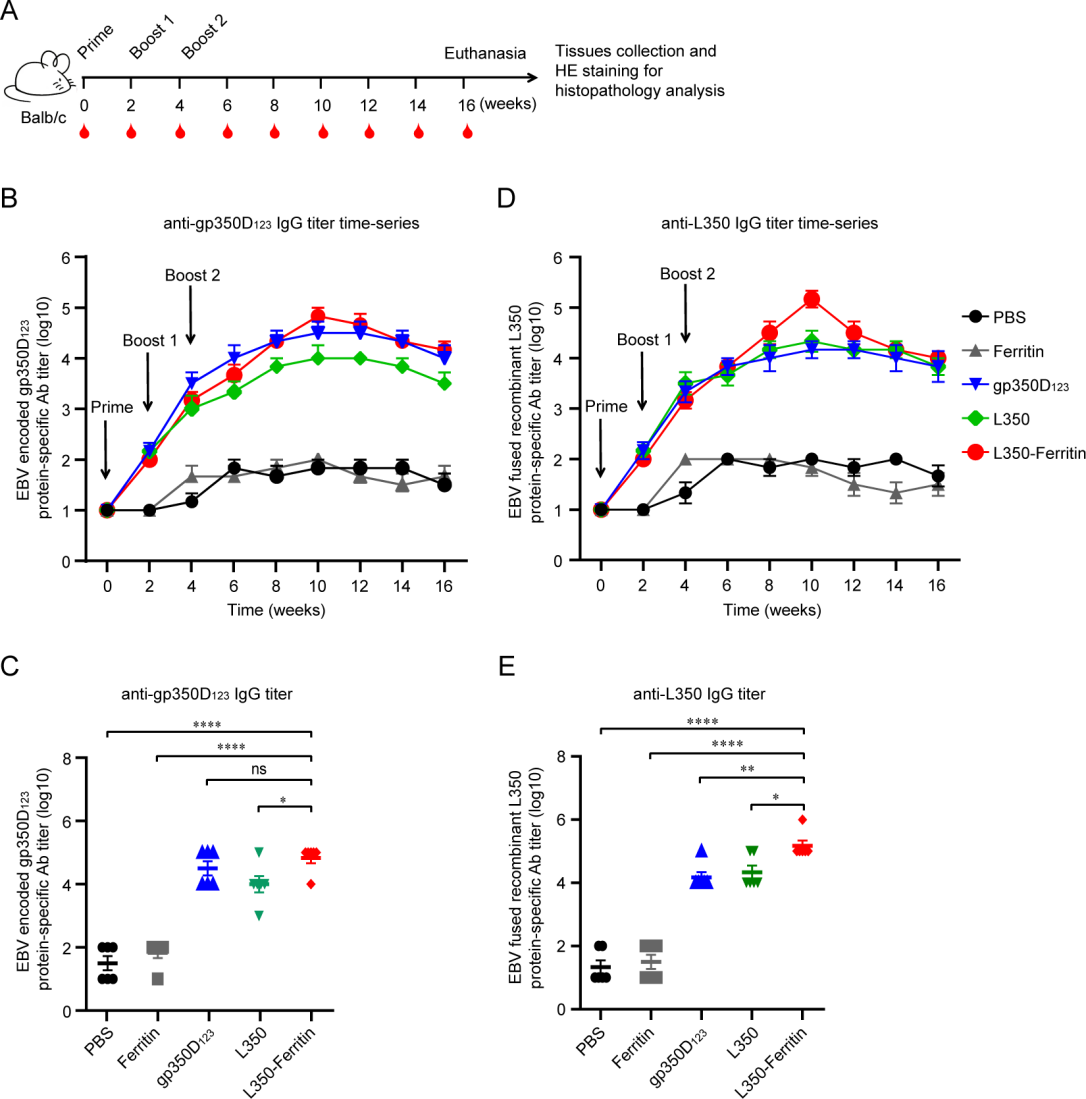
Immunogenicity of the nanoparticle vaccines in Balb/c mice. **(A)** Schematic of Balb/c mice vaccination. Six mice from each group were vaccinated with different vaccines at week 0 (prime), week 2 (boost 1) and week 4 (boost 2). Serum samples were collected every 2 weeks. All mice were euthanized at week 16 and the major tissues including heart, liver, spleen, lungs, and kidneys were collected for histopathology analysis, and the spleen of each immunized mice was collected for splenocyte isolation. **(B, C)** The gp350D_123_ protein-specific IgG titers **(B)** and recombinant L350 protein-specific IgG titers **(C)** of immunized Balb/c mice at each time point were detected by ELISA. IgG antibody titers of these serum samples were determined by serial dilution. **(D, E)** The gp350D_123_ protein-specific IgG titers **(D)** and recombinant L350 protein-specific IgG titers **(E)** of immunized Balb/c mice at week 10 were displayed. *n* = 6 biological replicates. Data are mean ± SD. Two-tailed unpaired Student’s *t-*test was used for statistical analysis. *p < 0.05, **p < 0.01, ****p < 0.0001 and n.s., not significant.

Balb/c mice were immunized with the L350–ferritin nanoparticle vaccine to assess its immunogenicity, and the mice injected with PBS, ferritin nanoparticles, L350 protein, and gp350D_123_ protein, expressed and purified from 293F cells (**Supplementary Figure S1**), served as controls. All these groups were immunized with proteins in an equimolar mass manner. These mice were initially vaccinated at week 0, and two booster injection were given at 2-week intervals (weeks 2 and 4). The serum samples of immunized mice were collected at weeks 0, 2, 4, 6, 8, 10, 12, 14, and 16 for serological testing (**Figure 4A**). The gp350D_123_ protein-specific and L350 protein-specific IgG titers of these sera samples were measured by enzyme-linked immunosorbent assay (ELISA) (**Figures 4B–E**). As shown in **Figures 4B, C**, after two rounds of booster immunizations, the L350 protein-specific and gp350D_123_-specific antibody titers reached their peak at week 10 in all immunized groups. Interestingly, the L350–ferritin nanoparticle and L350 protein could induce high titer of the gp350D_123_-specific antibody (**Figure 4B**), indicating that these epitopes in the fused recombinant L350 protein were fully exposed *in vivo*. Furthermore, the gp350D_123_-protein also induced high titers of L350 protein-specific antibody (**Figure 4C**), indicating that these epitopes in gp350 protein were indeed accessible *in vivo*. The gp350D_123_ protein-specific and L350 protein-specific antibody titers of week 10 in **Figures 4D, E** showed that the L350–ferritin nanoparticle elicited higher antigen-specific antibodies than L350 protein. These data indicated that the L350–ferritin nanoparticle vaccine could successfully induce potent antibodies to recognize EBV-encoded gp350 protein.

### The L350–ferritin nanoparticle vaccine elicited potent production of neutralizing antibodies

**Figure 5:**
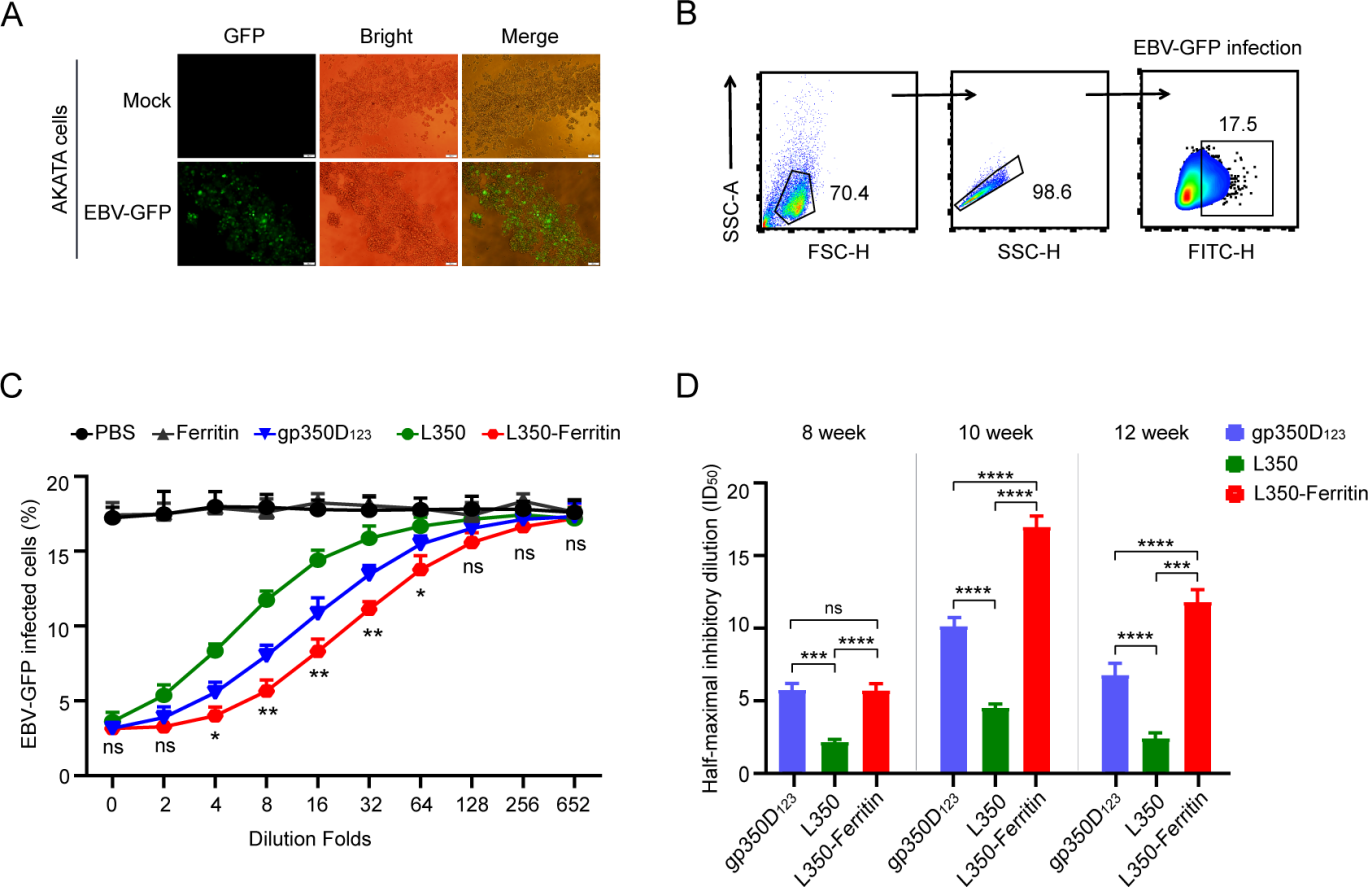
Neutralization of EBV infection. **(A)** AKATA cells were used to detect the infection of purified EBV-GFP reporter virus. Approximately 48 h postinfection, the infected cells were observed by GFP fluorescence. Scale bars represent 100 μm. **(B)** Gating strategy for detecting EBV-GFP-infected AKATA cells. Flow cytometry was used to analyze the proportion of GFP+ cells. **(C, D)** Neutralization of EBV-GFP infection by sera from vaccinated Balb/c mice. Serially diluted sera samples collected at week 10 were used to block EBV-GFP infection in AKATA cells, and the percentage of infected cells were detected and the significances between the gp350ECD_123_ protein group and the L350–ferritin nanoparticle group were calculated **(C)**. The half-maximal inhibitory dilution (ID_50_) of sera from vaccinated Balb/c mice at weeks 8, 10, and 12 was investigated **(D)**. *n* = 6 biological replicates. Data are mean ± SD. Two-tailed unpaired Student’s *t-*test was used for statistical analysis. *p < 0.05, **p < 0.01, ***p < 0.001, ****p < 0.0001 and n.s., not significant.

After confirming that the vaccine generated high levels of functional antibodies, we further assessed the neutralizing ability of these sera samples from immunized mice. Firstly, the EBV-GFP reporter virus, carrying the GFP gene in genome, was produced from CNE2-EBV-GFP cells and purified by ultracentrifugation. AKATA cells were infected with the purified EBV-GFP virus, and these cells showed strong GFP fluorescence at 48 h post-infection (**Figure 5A**), confirming the capability of EBV-GFP virus in infecting AKATA cells. Flow cytometry analysis of GFP-positive cells determined an approximate infection rate of 17.5% of the EBV-GFP reporter virus solution (**Figure 5B**). Subsequently, the sera samples from immunized mice at week 10 were serially diluted to detect the blocking efficiency of EBV-GFP viral infection in AKATA cells. As shown in **Figure 5C**, sera samples from L350–ferritin nanoparticle vaccine-immunized mice showed stronger neutralizing efficiency compared to other immunized mice, and these sera samples from L350 protein-immunized mice showed lower neutralizing efficiency compared to gp350D_123_ protein-immunized mice. The ID_50_ values of the sera samples of L350 protein, gp350D_123_ protein, and L350–ferritin nanoparticle vaccine at week 10 were 4.53, 10.03, and 16.98, respectively. The ID_50_ values of these sera samples at week 8, week 10, and week 12 were calculated, and the L350–ferritin nanoparticle vaccine was significantly stronger than others at week 10 and week 12 (**Figure 5D**). These data indicated that the L350–ferritin nanoparticle vaccine could induce potent neutralizing antibody titers in immunized mice.

### The L350–ferritin nanoparticle vaccine induced strong antigen-specific B-cell memory

**Figure 6:**
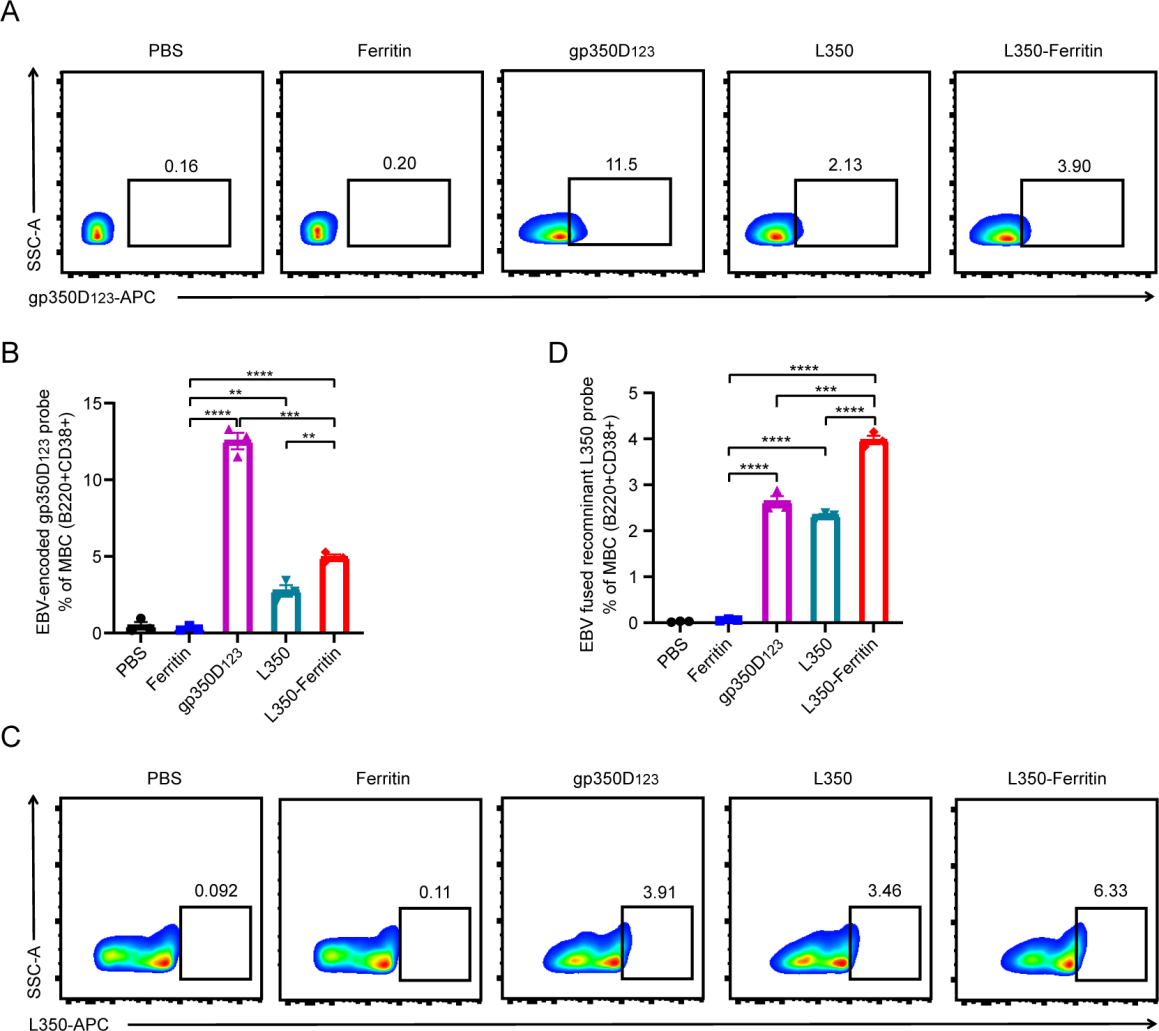
Immunological B-cell memory in vaccinated mice. **(A–D)** The gp350D_123_ protein **(A)** and recombinant L350 protein **(C)** were labeled with NHS-APC and purified to incubate with the splenocytes of vaccinated mice, and the fluorescence-conjugated monoclonal antibodies (B220-PE, CD38-FITC) were co-incubated with them to stain memory B cell (B220+/CD38+), and then the APC+/B220+/CD38+ B cells were detected by flow cytometry. The percentage of the gp350D_123_ protein-specific memory B cells **(B)** and recombinant L350 protein-specific memory B cells **(D)** were calculated. *n* = 3 biological replicates. Data are mean ± SD. Two-tailed unpaired Student’s *t-*test was used for statistical analysis. **p < 0.01, ***p < 0.001, ****p < 0.0001.

To investigate the antigen-specific memory B-cell response in immunized mice induced by the L350–ferritin nanoparticles vaccine, the splenocytes of mice were collected at week 16. The gp350D_123_ protein and L350 protein were labeled with APC fluorescence, and incubated with splenocytes. The memory B cells (MBCs) in splenocytes were classified and marked by anti-B220 and anti-CD38 antibodies. As shown in **Figure 6**, the L350–ferritin nanoparticle vaccine generated more gp350D_123_ protein-specific MBCs (gp350D_123_+/B220+/CD38+) and L350 protein-specific MBCs (APC+/B220+/CD38+) than L350 protein monomers. However, in gp350D_123_ protein-immunized mice, the gp350D_123_ protein-specific MBCs (gp350D_123_+/B220+/CD38+) were higher than other groups, which could be involved in the multiple epitopes on the surface of the gp350 protein. Interestingly, the L350–ferritin nanoparticle vaccine-induced MBCs could effectively recognize the gp350D_123_ protein and L350 protein, which was consistent with the results showing that the L350–ferritin nanoparticle vaccine induced high titers of gp350D_123_ protein-specific and L350 protein-specific antibody. Based on these findings, we concluded that the L350–ferritin nanoparticles vaccine could provide persistent protective humoral responses.

### The L350–ferritin nanoparticle vaccine demonstrates favorable safety in Balb/c mice

**Figure 7:**
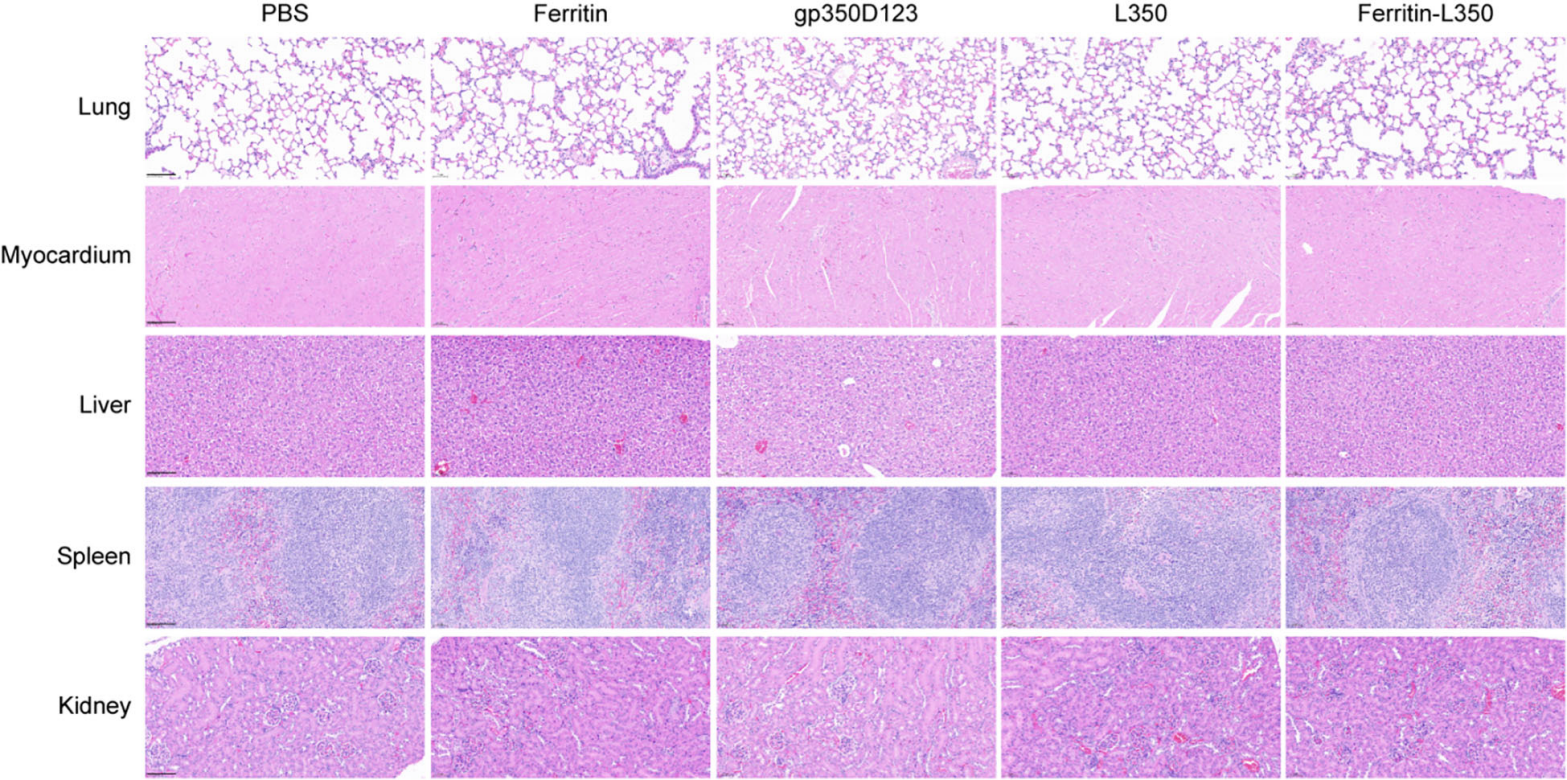
Histopathological analysis of tissues from vaccinated mice. Tissues from vaccinated mice were stained by HE, including lungs, myocardium, liver, spleen, and kidney. Scale bars represented 100 μm.

At the conclusion of the study, the histopathological analysis of tissues including lungs, heart, liver, spleen, and kidneys from vaccinated mice was performed (**Figure 7**). The histological sectioning and H&E staining showed no significant histopathological changes in these tissues, and none of the inter-group differences were observed, indicating that the L350–ferritin nanoparticle vaccine exhibited favorable safety.

## Discussion

Vaccines have been demonstrated as one of the safest and most effective methods for preventing infectious diseases and pathogens. EBV is a significant oncogenic virus with high associations with various malignancies; however, no vaccines specifically targeting EBV have been approved to date. Subunit vaccines based on gp350 glycoprotein were widely studied, and they were reported to effectively elicit specific binding and neutralizing antibodies (34, 35). In this study, we selected five linear neutralizing epitopes of EBV-encoded gp350 glycoprotein to design a fused recombinant antigen (L350) and then bound them on the surface of *H. pylori*-derived ferritin nanoparticle to develop a nanoparticle vaccine. This vaccine induced robust gp350 protein-specific and L350 protein-specific antibodies and elicited significantly higher titers of neutralizing antibodies in immunized Balb/c mice, compared with other controls. Additionally, the L350–ferritin nanoparticle vaccine induced higher gp350D_123_ protein-specific and L350 protein-specific memory B cells, suggesting its capability to establish longer-lasting immune memory and humoral immunity to counter potential EBV infections over the course of life.

The gp350 glycoprotein was reported to be covered by high-density glycans (18), providing difficulty for vaccine design. The epitopes without glycan on gp350 glycoprotein mediated its binding with the receptor CD21/CR2 protein (19–21). Therefore, vaccines composed of these epitopes might be more efficient than the full-length gp350 glycoprotein. However, in most cases, a single linear peptide is insufficient to induce a robust antibody response to prevent viral invasion. Peptide antigens do not elicit antibodies against conformation-dependent epitopes, making linear peptides less immunogenic than larger protein antigens, a potential inherent limitation. These epitope-based vaccines were limited by their low molecular weight to induce lower immunogenicity. In this study, we fused these epitope peptides with flexible linkers and displayed the recombinant antigens on the surface of ferritin self-assembled nanoparticles, which increased the molecular weight of antigens. The antigen-specific antibody and neutralizing antibody induced by nanoparticle vaccine in this study showed a significant increase in immunized mice, compared with monomeric antigen, indicating that the ferritin nanoparticle promoted the immunogenicity of the epitope-based antigens.

The ferritin nanoparticle is well-studied in vaccine research (14, 15, 36). The 24-mer ferritin core could induce ferritin-specific antibody, but prior studies have shown that the titers of virus-specific antibodies were not significantly affected, such as antibodies against SARS-CoV-2 and influenza (37, 38). Ferritin from *H. pylori* and its corresponding antibodies demonstrated low toxicity *in vivo*, and several ferritin-based influenza nanoparticle vaccines have been completed or are undergoing clinical evaluation (NCT03186781 and NCT03814720), without reports of serious adverse effects, indicating the promising application of ferritin nanoparticle in vaccine development.

Safe and effective vaccines that address EBV infections or latent infections are in urgent demand. Here, we reported a nanoparticle vaccine based on epitope peptides from the EBV-encoded gp350 protein. This vaccine showed remarkable immunogenicity, strong neutralizing antibody responses and memory B cells, and also potential safety. The EBV-encoded gp350 protein has consistently been the primary immunogen for vaccine development, though antibodies against it mainly prevent B-cell infection. EBV also encodes dozens of envelope glycoproteins, and all of them can be tested as antigens in vaccines. These nanoparticles, such as ferritin polymers, chimeric VLPs (27), and porous dodecahedral i301 (39), provided platforms for multiple envelope glycoproteins displaying. In future studies, high-quality epitopes of other envelope glycoproteins, such as gL, gH, gp42, and gB proteins, should be investigated to determine the combination of multiple antigens in nanoparticle vaccines that will provide promising prophylactic vaccines for EBV prevention.

## Data Availability

The original contributions presented in the study are included in the article/**Supplementary Material**. Further inquiries can be directed to the corresponding authors.
