# Corrigendum: A self-assembled nanoparticle vaccine elicits effective neutralizing antibody response against EBV infection

**DOI:** 10.3389/fimmu.2025.1559927

**Published:** 2025-02-04

**Authors:** Ping Li, Ziyi Jiang, Jingjing Shi, Haochuan Sha, Zihang Yu, Yan Zhao, Sanyang Han, Lan Ma

**Affiliations:** ^1^ Institute of Biopharmaceutical and Health Engineering, Tsinghua Shenzhen International Graduate School, Tsinghua University, Shenzhen, China; ^2^ Institute of Biomedical Health Technology and Engineering, Shenzhen Bay Laboratory, Shenzhen, China; ^3^ College of International Education, Henan University of Technology, Zhengzhou, China; ^4^ Institute of Bio-Architeture and Bio-Interactions, Shenzhen Medical Academy of Research and Translation, Shenzhen, China; ^5^ State Key Laboratory of Chemical Oncogenomics, Tsinghua Shenzhen International Graduate School, Tsinghua University, Shenzhen, China

**Keywords:** Epstein-Barr virus (EBV), vaccine, epitope, ferritin, nanoparticle

## Error in Figure/Table

In the published article, there was an error in **Figure 7** Histopathological analysis of tissues from vaccinated mice as published. We misused an incorrect image of mouse myocardium in the PBS group (Column 1, Row 2) during the selection from our extensive dataset. The corrected **Figure 7** Histopathological analysis of tissues from vaccinated mice and its caption Tissues from vaccinated mice were stained by HE, including lungs, myocardium, liver, spleen and kidney. Scale bars represented 100 μm. appear below.

In the published article, there was another error in **Figure 3** Characterization of self-assembly of the fusion antigens into ferritin nanoparticles as published. In **Figure 3B**, we misused the image of Ferritin nanoparticles (1^st^ and 3^rd^ images, Left panel)) during the selection from our extensive dataset. The corrected **Figure 3** Characterization of self-assembly of the fusion antigens into ferritin nanoparticles and its caption (A) Dynamic light scattering (DLS) analyzing the particle size of the purified ferritin nanoparticles and L350-Ferritin nanoparticles. The curves of the particle size were drawn by GraphPad Prism 8.3 software. Ferritin nanoparticles (Blue), L350-Ferritin nanoparticles (Red). n = 3 independent repeats. Data are mean ± SD. (B) Transmission electron microscopy (TEM) analyzing the shape of the purified ferritin nanoparticles and L350-Ferritin nanoparticles. Scale bars represent 100 nm (upper) and 20 nm (Lower) appear below.

**Figure 3:**
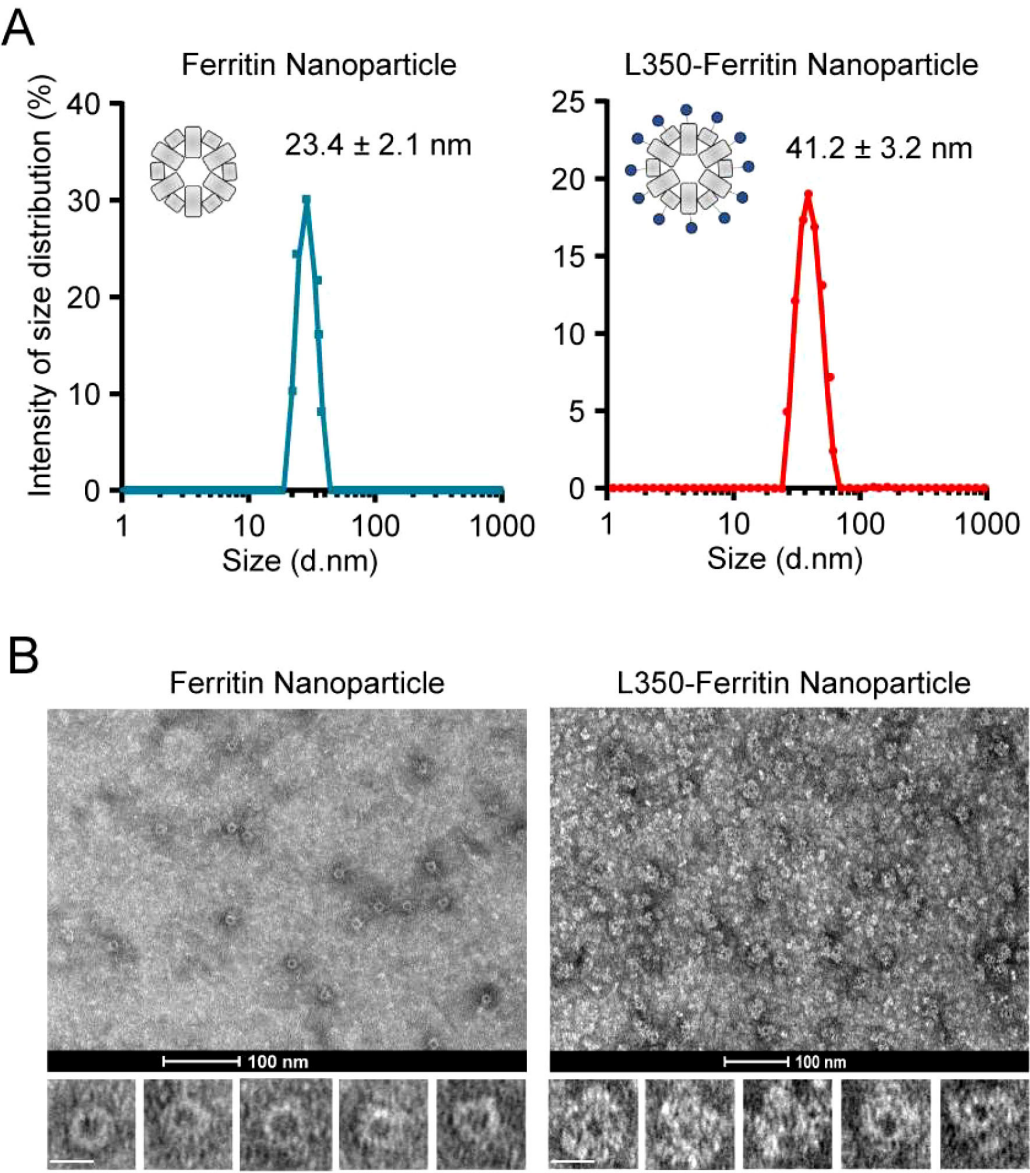
Characterization of self-assembly of the fusion antigens into ferritin nanoparticles. **(A)** Dynamic light scattering (DLS) analyzing the particle size of the purified ferritin nanoparticles and L350-Ferritin nanoparticles. The curves of the particle size were drawn by GraphPad Prism 8.3 software. Ferritin nanoparticles (Blue), L350-Ferritin nanoparticles (Red). n = 3 independent repeats. Data are mean ± SD. **(B)** Transmission electron microscopy (TEM) analyzing the shape of the purified ferritin nanoparticles and L350-Ferritin nanoparticles. Scale bars represent 100 nm (upper) and 20 nm (Lower).

**Figure 7:**
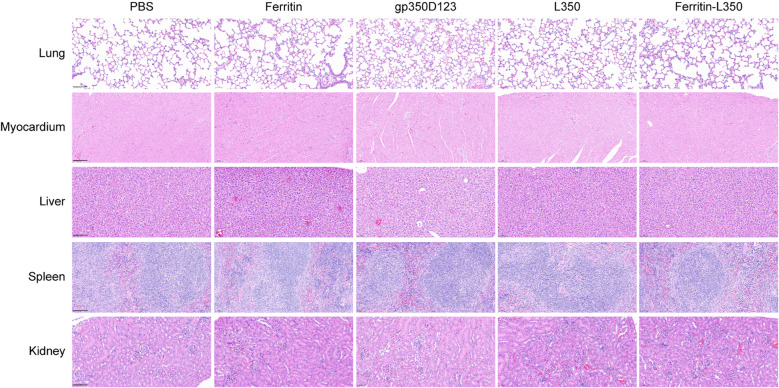
Histopathological analysis of tissues from vaccinated mice. Tissues from vaccinated mice were stained by HE, including lungs, myocardium, liver, spleen and kidney. Scale bars represented 100 μm.

The authors apologize for this error and state that this does not change the scientific conclusions of the article in any way. The original article has been updated.

