# Supplementary material for: A self-assembled nanoparticle vaccine elicits effective neutralizing antibody response against EBV infection
Source: Front Immunol. 2025 Jan 3;15:1530364. doi: 10.3389/fimmu.2024.1530364 (PMC11739326; doi:10.3389/fimmu.2024.1530364)
Supplement: Supplementary file 1 [file DataSheet1.docx]

**Supporting Information for**

A self-assembled nanoparticle vaccine elicits potent neutralizing antibody response against EBV infection

Ping Li^a,b,#^, Ziyi Jiang^a,#^, Jingjing Shi^a,#^, Haochuan Sha^c^, Zihang Yu^d^, Yan Zhao^b^, Sanyang Han^a,^*, Lan Ma^a,b,e,^*

^a^*Institute of Biopharmaceutical and Health Engineering, Tsinghua Shenzhen International Graduate School, Tsinghua University, Shenzhen, 518055, China.*

*^b^Institute of Biomedical Health Technology and Engineering, Shenzhen Bay Laboratory, Shenzhen, 518132, China.*

*^c^College of International Education, Henan University of Technology, Zhengzhou, 45000, China.*

*^d^Institute of Bio-Architeture and Bio-Interactions, Shenzhen Medical Academy of Research and Translation, Shenzhen, 518132, China.*

*^e^State Key Laboratory of Chemical Oncogenomics, Tsinghua Shenzhen International Graduate School, Tsinghua University, Shenzhen, 518055, China*

^#^These authors contributed equally to this work.

**Corresponding author. Institute of Biopharmaceutical and Health Engineering, Tsinghua Shenzhen International Graduate School, Tsinghua University, University Town of Shenzhen, Nanshan District, Shenzhen, 518055, China.*

Lan Ma: *malan@sz.tsinghua.edu.cn;* Sanyang Han: *hansanyang@sz.tsinghua.edu.*

**Supplementary figures and figure legend**

**Fig S1. Identification of gp350_D123_ protein.** The gp350D_123_ protein purified from 293F cells was detected by SDS-PAGE with coomassie blue staining and Western-Bloting with gp350 protein specific antibody.

**
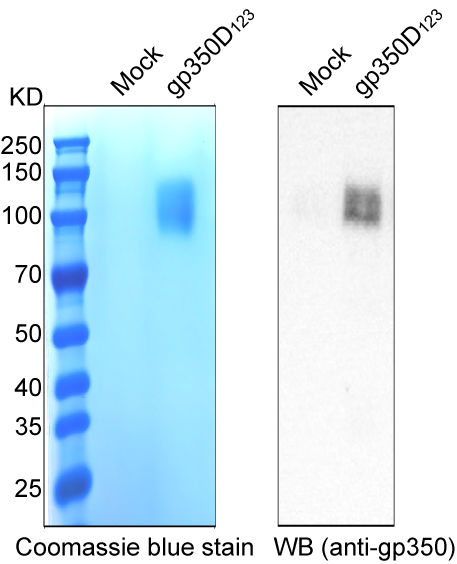
**
